# The parents voice on oral health care from two paediatric oncology units

**DOI:** 10.1007/s40368-026-01205-y

**Published:** 2026-04-05

**Authors:** J. Janeiro, T. Pinto, F. Leite, A. P. Fernandes, R. M. Silva, P. Correia

**Affiliations:** 1 Faculty of Dental Medicine, Universidade Católica Portuguesa, Viseu, Portugal; 2 Escuela de Doctorado en Cirugía y Odontoestomatología, University of Salamanca, Salamanca, Spain; 3 Portuguese Oncology Institute of Porto (IPO Porto), Porto, Portugal; 4 Paediatric Oncology, Centro Hospitalar Universitário São João, Porto, Portugal; 5 Center for Interdisciplinary Research in Health (CIIS), Universidade Católica Portuguesa, Viseu, Portugal; 6 Faculty of Health Sciences, Fernando Pessoa University, Porto, Portugal; 7 Rise-Health, Faculty of Medicine, University of Porto, Porto, Portugal

**Keywords:** Oral care, Oral complications, Cancer, Oncology units, Children

## Abstract

**Purpose:**

Oral complications, such as oral mucositis, are amongst the most frequent side effects of antineoplastic therapy, particularly in children. Maintaining adequate oral care and managing oral mucositis can be challenging. Parents are required to support their child during cancer treatment but often lack confidence and knowledge. The present study aims to determine whether parents received information on oral hygiene instructions during their child’s cancer treatment and to assess the provision of oral care before, and after cancer diagnosis.

**Methods:**

A cross-sectional observational study was performed. A questionnaire was applied to a convenience sample of parents of children diagnosed with cancer, at two Paediatric Oncology Units in the North of Portugal.

**Results:**

A total of 166 parents participated (IPO-Porto: 104; CHUSJ: 62). Approximately 58% (*n* = 96) of the children had regular dental examinations before cancer diagnosis; 87% (*n* = 144) of the parents were informed about potential oral complications before cancer treatment and 85% (*n* = 141) of the children presented oral complications during and after treatment particularly mouth ulcers/thrush (88% in both hospitals). During cancer treatment, 60% of children from IPO-Porto and 49% from CHUSJ brushed their teeth twice a day. Most parents highly valued the role of dentists as integral members of the multidisciplinary medical team supporting their children during cancer treatment.

**Conclusion:**

The present findings highlight that oral care instructions provided to parents during cancer treatment were insufficient at both Paediatric Oncology Units, although parental engagement was high. Reported dental care before and after diagnosis was below recommended clinical guidelines.

## Introduction

Despite paediatric cancer generally having a better prognosis than adult cancer, children with cancer and their families still experience a considerable burden on their quality of life and well-being during treatment. Following a child’s cancer diagnosis, parents require support to understand and implement the information and guidance provided to them (Keith et al. 2023; Tan et al. 2024).

Health literacy can be defined as the ability to meet knowledge needs in order to reduce anxiety or fear, inform decision-making, and support the acquisition of relevant skills (Urstad et al. 2022). Following a cancer diagnosis, parents are entitled to receive comprehensive information about their child’s care needs; however, oral health is frequently overlooked during these challenging and distressing times (McKenna et al. 2010; Sajwani et al. 2024).

In spite of the significant developments in cancer treatment, chemotherapy is still the most common treatment modality for various types of cancer and is associated with many side effects (Markert et al. 2009). Along with nausea and vomiting, oral mucositis (OM) is one of the most frequent side effects associated with the treatment of paediatric patients (Attinà et al. 2021), regardless of the type of cancer (Sonis 2004). Therefore, maintaining good oral care is essential in children with cancer, due to the risk of oral-related infections and increased susceptibility to general adverse effects particularly during the periods of immunosuppression (de Lima Martins et al. 2023; Docimo et al. 2022).

Chemotherapy regimens are typically administered in cycles, and oral complications, such as OM, commonly develop around five to eight days after the initiation of each cycle, coinciding with a reduction in neutrophils, platelets, and other white blood cells (Ritwik 2018; Pulito et al. 2020).

Oral mucositis is a debilitating condition characterised by inflammation of the oral mucosa induced by antineoplastic therapies. Its incidence is higher in paediatric patients, ranging from 40 to 100%, compared to adults (Allen et al. 2010; Braguês et al. 2024). In its severe form, OM presents with extensive mucosal ulceration, necrosis, and spontaneous bleeding, substantially impairing the patient’s quality of life (Ward et al. 2015; Barkokebas et al. 2015). Moreover, OM can influence both the duration and intensity of cancer treatment. Severe cases may necessitate enteral or parenteral nutritional support, and in some instances, anticancer therapy must be reduced or suspended, potentially compromising treatment outcomes and prognosis (Braguêss et al. 2024).

Current guidelines advise an initial dental appointment, prior to cancer treatment, to establish an adequate oral treatment plan, best suited for the child's condition, alongside regular assessments of the mouth during treatment. Patients who have received dental treatment and oral health instructions, prior to starting cancer therapy, show a significantly lower incidence of oral mucositis (Chaveli-López and Bagán-Sebastián, 2016). The implementation of oral health education programs is now known to be an important measure to prevent the development of OM. The Multinational Association of Supportive Care in Cancer (MASCC) added patient education as a new intervention category to prevent OM lesions (Bezerra et al. 2022). Parents should receive adequate information on assisting their child’s routine oral healthcare, as well as advice on the most common oral complications, such as OM (Tan et al. 2024; Ritwik 2018; Allen et al. 2010; Chaveli-López and Bagán-Sebastián, 2016).

The aim of the present study was to evaluate if parents were informed about oral health and the oral hygiene practices of their children before, during, and after cancer treatment in two paediatric oncology units in Portugal. IPO-Porto and CHUSJ, centres of reference in paediatric oncology in the North region of Portugal, maintain a strategic partnership, whith IPO-Porto primarily managing liquid tumours and CHUSJ solid tumours. Despite this collaboration, variations in oral care protocols exist between the institutions. Consequently, this study includes both units to explore how these differences influence patient communities and their oral health perceptions.

## Material and methods

### Study design, population and sample

A cross-sectional observational study was conducted on a convenience sample of parents of children receiving treatment for cancer in two hospitals located in the city of Porto, in the north of Portugal: the Paediatric Oncology Service of the Centro Hospitalar Universitário de São João (CHUSJ) and the Instituto Português de Oncologia do Porto Francisco Gentil (IPO-Porto). A questionnaire was utilized to collect sociodemographic, clinical, and other relevant data between 2019 and 2020.

This study obtained approval from the Ethics Committee for Health at the CHUSJ (authorization number: 438/19), the Ethics Committee for Health at the IPO-Porto (authorization number: 48/019) and the Ethics Committee for Health at the Catholic University of Portugal (CES-UCP) (authorization number: 39).

In both hospitals, the study participants were the primary caregivers and legal guardians of the child who accompanied the child to the hospital. The inclusion criteria comprised children with inpatient or outpatient status, aged up to 18 years, and undergoing active treatment (any period between early treatment and completion of active therapy) or in cancer remission. The exclusion criteria included children with an unclear diagnosis or who did not have cancer therapy, as advised by medical professionals.

### Ethical aspects

Written informed consent was obtained from the parents. All collected data were stored and processed, according to the Helsinki Declaration of the World Medical Association and the General Data Protection Regulation (Regulation (EU) 2016/679).

### Data registration and reporting

The questionnaire was developed *de novo* by the authors and subsequently underwent linguistic validation to ensure clarity and cultural appropriateness for the target population.

The questionnaire included sociodemographic questions (age, sex, kinship, parent’s educational level, medical diagnosis and treatment duration), knowledge and practices of oral hygiene, and knowledge of the oral side effects during cancer treatment and parental satisfaction over the information and the dental care received. The report of the findings followed the STROBE checklist (von Elm et al. 2008).

### Data analysis

The statistical analysis of the data was carried out using the Statistical Package for the Social Sciences—SPSS for Windows (version 24.0; IBM, IL; USA).

To describe and characterise the sample, a descriptive and inferential analysis of the data was performed. For comparisons between the data from the Paediatric Oncology Service of CHUSJ and the IPO-Porto Oncology Service, the chi-square test and Fisher’s exact test were used. When required, the statistical significance level used in the present study was 5%.

## Results

At the IPO-Porto, 104 parents were recruited, with a response rate of 100%. Most tumours were liquid tumours (78%) followed by solid tumours (19%).

The study at the CHUSJ had a response rate of 95% and included 62 parents. Cancer diagnosis varied between central nervous system (CNS) solid tumours (55%) and other solid tumours (45%). The prevalence of tumour types between IPO-Porto and the CHUSJ is compared in Table 1.

**Table 1:** Types of malignant tumours at IPO-Porto and CHUSJ

Type of tumour	IPO-Porto		CHUSJ	
	n	%	n	%
Liquid Tumours	81	78%	–	–
Solid Tumours CNS	3	3%	18	55%
Other Solid Tumours	20	19%	15	46%
Total	104	100%	33	100%

At IPO-Porto 89% of children presented with oral complications. At CHUSJ the number was lower, 73%. This difference was statistically significant (*p* = 0.025).

The demographic distribution indicated that at IPO-Porto, most patients were female (53%, *n* = 55) and tended to be aged 10 years or older. At CHUSJ, most patients were male (55%, *n* = 18) and most were between 6 and 10 years of age.

Approximately half of the children from both hospitals had routine dental appointments before being diagnosed with cancer (Table 2). Dental appointments after cancer diagnosis were associated with dental attendance prior to cancer diagnosis (*p* < 0.001).

**Table 2:** Dental appointments before and after cancer diagnosis at IPO-Porto and CHUSJ

		IPO-Porto	CHUSJ
Routine dental appointments before diagnosis	No	35%	55%
	Yes	65%	46%
Routine dental appointments after cancer diagnosis	No	39%	52%
	Yes	62%	49%
OH instructions after cancer diagnosis	No	15%	24%
	Yes	85%	76%

Most caregivers reported receiving oral health instructions following cancer diagnosis, 85% at IPO-Porto and 76% at CHUSJ. Around 30% of the caregivers from IPO-Porto and 41% from CHUSJ received instructions from the medical team. The nursing team also provided oral health instructions, at IPO-Porto 47%, and at CHUSJ 18%. At IPO-Porto 23% of the caregivers reported that oral health instructions were provided by dentists compared to 41% of parents from CHUSJ (Table 3).

**Table 3:** Oral health instructions and oral hygiene practices at IPO-Porto and CHUSJ

Variable	IPO-Porto n (%)	CHUSJ n (%)
Instructions from medical team	31 (30%)	25 (41%)
Instructions from nursing team	49 (47%)	11 (18%)
Instructions from dentists	24 (23%)	25 (41%)
Brushes teeth twice/day	62 (60%)	30 (49%)
Complete OH (brush + floss + mouthwash)	4 (4%)	4 (4%)

Considering oral hygiene routines at IPO-Porto and CHUSJ, most of the children had adequate toothbrushing frequency (Table 3).

Regarding the amount of fluoride in the child’s toothpaste, most caregivers could not recall the percentage of fluoride. From those who did (27%), 9% used toothpaste with a low percentage of fluoride or fluoride-free toothpaste.

Most caregivers from both hospitals felt uncomfortable using dental floss. Complete oral hygiene care that included toothbrushing with fluoride toothpaste, dental floss and mouthwash was performed by 3.6% of the children at both hospitals (Table 3).

**Fig. 1:**
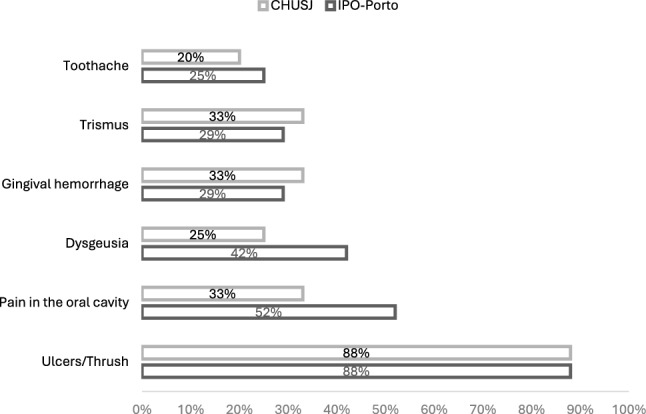
Parental recall of oral complications presented by children at IPO-Porto and CHUSJ during cancer treatment

Most of the caregivers (79%) at both hospitals reported being informed about possible oral complications due to cancer treatment. At the IPO-Porto, 89% of the children had oral complications related to cancer treatment. Among these children, approximately 88% presented with ulcers or oral thrush (candidiasis), 52% experienced oral pain, 42% reported dysgeusia (taste alterations), 29% exhibited gingival bleeding and trismus (difficulty opening the mouth), and 25% reported toothache. At CHUSJ, 73% of the children presented with oral complications. Of these, 88% had ulcers/thrush during cancer treatment, 33% had associated pain in the oral cavity, gingival haemorrhage and trismus, 25% had dysgeusia, and 20% of the children reported toothache. The remaining oral manifestations presented by children from both hospitals had an overall frequency under 20% (Fig. 1).

**Fig. 2:**
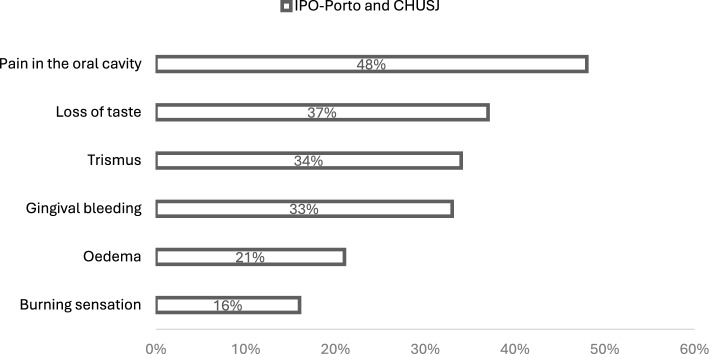
Parental recall of multiple oral manifestations associated with ulcers/thrush in children from IPO-Porto and CHUSJ

The co-occurrence of ulcers/thrush in the oral cavity with other oral manifestations was also evaluated. From those who presented ulcers/thrush (88%), 48% complained of pain in the oral cavity, 37% reported loss of taste, 34% reported difficulties in opening their mouths, 33% presented gingival bleeding, 21% had oedema and 16% complained of a burning sensation in both hospitals (Fig. 2).

Approximately 20% of caregivers sought dental care only when the child had a complaint, while 26% reported no dental follow-up during cancer treatment. Regarding the importance of including a dentist on the hospital team, 97% of caregivers from IPO-Porto and 91% from CHUSJ acknowledged its value.

At IPO-Porto, 51% of caregivers were satisfied with the oral care received, compared to 68% at CHUSJ.

## Discussion

Oral complications in children undergoing cancer treatment are common and debilitating side effects, significantly impacting patients’ quality of life. Improving parents’ knowledge and management skills, particularly regarding OM and other therapy-related complications, is therefore crucial (Ward et al. 2015; Barkokebas et al. 2015). As primary caregivers, parents play a pivotal role in maintaining their child’s oral hygiene, alleviating symptoms, and advocating for their overall well-being (Gan et al. 2025). Understanding parental knowledge gaps and unmet needs are fundamental to guide clinicians and healthcare institutions in developing educational programs and family-centred support systems (Keith et al. 2023).

The present study aimed to assess the information parents receive regarding oral care required during their child’s cancer treatment, as well as routine oral hygiene practices, through a questionnaire administered at two Paediatric Oncology Units in the North of Portugal: the Paediatric Oncology Services of CHUSJ and IPO-Porto. Both units provide comprehensive outpatient and inpatient care with dedicated stomatology departments that monitor paediatric cancer patients before, during and after cancer treatment.

At IPO-Porto, information about oral hygiene practices and potential oral complications following a cancer diagnosis was primarily provided by the nursing team. In contrast, at CHUSJ, this information was delivered by either the medical team or dentists, typically during private appointments. These observations align with existing literature, which indicates that oral health information is often conveyed by the healthcare professionals in closest contact with the patient, such as nursing or medical oncology teams (Glenny et al. 2004; Keith et al. 2023). The inclusion of qualified oral health professionals, such as dentists or stomatologists, within a multidisciplinary oncology team is critical for several reasons. Regular monitoring of a patient’s oral health by these specialists not only benefits the child, by reducing the incidence and severity of complications (e.g., mucositis, infection), but also enhances the overall efficiency and quality of care (Sajwani et al. 2024; Blakemore 2024).

Non-adherence to dental treatments and routine oral hygiene among paediatric cancer patients increases the risk of developing oral complications during cancer treatment. Ideally, a comprehensive dental assessment should occur prior to the initiation of cancer treatment to mitigate or eliminate risk factors for infection, pain, or other oral health issues. Such consultations also provide an opportunity to educate both children and parents on preventive strategies and symptom management (Blakemore 2024; Bezerra et al. 2021). This is particularly critical in paediatric oncology, where oral complications, such as mucositis, infections and xerostomia, can severely impact treatment tolerance and quality of life.

Understanding the benefits of good oral hygiene in preventing treatment-related oral complications is critical for motivating both parents and children (Glenny et al. 2004). In the present study, nearly all parents valued the presence of a dentist as part of the hospital medical team, indicating a strong commitment to their child's oral health management during cancer treatment.

Furthermore, the present findings reveal that approximately 83% of parents reported receiving information about oral hygiene after the cancer diagnosis, whilst 87% were informed about potential oral complications. Given that 85% of children experienced oral complications during treatment, it is essential to implement targeted strategies that empower parents with the knowledge, skills and confidence needed to provide effective and informed oral health support for their child's oral health needs.

The difference in the prevalence of oral complications between children treated at IPO-Porto and CHUSJ was statistically significant (p < 0.05), a finding that may reflect variations in the predominant cancer types treated at each institution. Specifically, patients with malignant haematological tumours, such as those predominantly managed at IPO-Porto, exhibit a higher prevalence of oral manifestations compared to children with solid tumours. This is likely attributable to the intensity and myelosuppressive nature of therapeutic regimens commonly used in haematological malignancies (Damascena et al., 2020).

In our sample, the most prevalent oral manifestations were ulcers and thrush, primarily associated with oral mucositis, followed by oral discomfort as the second most frequent oral complication. Dysgeusia was also commonly reported. These findings align with previous studies which highlight the high incidence of mucositis-related complications in paediatric oncology (Patel et al. 2021). Oral pain, frequently reported in our cohort, was predominantly linked to thrush and oral mucositis (Mosel et al. 2011). Dysgeusia, a recurrent side effect in cancer patients, arises due to the cytotoxic effect of chemotherapy and/or radiotherapy in the nerves and olfactory receptors, particularly during cellular renewal of the oral epithelium. In severe cases, this alteration in taste perception might become permanent (Mosel et al. 2011). Interestingly, trismus exhibited a lower prevalence amongst children treated at IPO-Porto compared to those at CHUSJ. This discrepancy may be attributed to differences in treatment regimens, patients with solid tumours, particularly those treated at CHUSJ, where brain tumours predominated, often undergo combined chemotherapy and radiotherapy, or radiotherapy alone (Andabak et al. 2017). Such regimens can induce lesions in the masticatory muscles, and reduce tissue blood flow, and promote connective tissue formation and muscle fibrosis, ultimately leading to trismus (Bensadoun et al. 2010). The higher prevalence of brain tumours at CHUSJ, which frequently require radiation to the head and neck region, likely contributes to this increased incidence prevalence of trismus (Bensadoun et al. 2010).

Toothache was reported by 25% of children monitored at IPO-Porto and 20% at CHUSJ, a finding that may be attributed to the neurotoxic effects of specific chemotherapy, such as methotrexate, which can induce neuropathic pain. While most oral complications usually resolve within a week after chemotherapy, tooth hypersensitivity may appear weeks, or even months, after treatment completion (Chaveli-López 2016).

Although most children in our sample exhibited oral manifestations, only 50% had dental appointments during cancer treatment. However, there was no statistically significant association between the prevalence of oral complications and access to dental care. Surprisingly, dental follow-ups did not correlate with the presence or severity of oral manifestations, suggesting that other factors, such as treatment intensity, individual susceptibility or parental adherence to oral care recommendations, may play a more critical role in determining oral health outcomes during cancer therapy.

Children's oral hygiene practices during cancer treatment were also assessed. Around 60% of parents stated their children brushed their teeth twice a day and almost every child used toothpaste with fluoride. This practice is in line with current oral health guidelines (Kumar et al. 2018; Elad et al. 2020; American Association of Pediatric Dentistry 2018). Dental floss use is reserved for children over the age of eight and typically under parental or caregiver supervision (Quinn 2020; Elad et al. 2020; American Association of Pediatric Dentistry 2018). This precaution is particularly relevant in paediatric oncology, where mucosal fragility and thrombocytopenia may increase the risk of bleeding or soft tissue bleeding. Mouthwashes, such as aqueous alcohol-free chlorhexidine gluconate and benzydamine HC, can serve as a valuable complement to oral hygiene during cancer treatment. They can help prevent outbreaks of infection in the oral cavity, can moisturise the oral mucosa, avoiding soft tissue trauma as well as changes in the pH, and can be used for symptom relief (Kumar et al. 2018).

The present study has some limitations, including the use of convenience samples from two hospitals within the same geographical region, and potential questionnaire bias due to reliance on participants’ recollection of events. The questionnaire recruited patients actively undergoing treatment (regardless of phase), which could add to sample heterogeneity due to the variability in cancer type, disease stage, as well as in treatment protocol and duration, and influence parental responses. Additionally, the questionnaire did not include the respondent profile and family sociodemographic data related to gender, age, parents’ education, income, among others. Given that dental care in Portugal is predominantly provided through the private sector, assessing the influence of access to dental services would have been relevant to determine the influence on oral health practices reported by parents (Patrick et al. 2006; Santos et al. 2024). Equally, it would have been relevant to obtain qualitative data, such as via a semi-structured interview to best assess parental concerns and suggestions.

Future research should explore the integration of routine dental assessments into the paediatric oncology admission protocol, including practical hands-on demonstrations of oral care techniques for parents and caregivers. Additionally, the creation of targeted oral health literacy resources, tailored to the needs of non-medical audiences, could empower parents to manage their child’s oral health more effectively, thereby improving quality of life and treatment adherence in this vulnerable population.

Oral health professionals, acting as part of the hospital’s multidisciplinary team, are critical to safeguarding oral health and optimising the management of treatment-related complications. With structured oral care follow-up, before, during, and after cancer therapy, many oral complications can be prevented or mitigated.

This study highlights a critical gap in paediatric oncology care: despite the known importance of oral health, access to consistent and equitable oral care remains insufficient for many children undergoing cancer treatment. Future efforts should focus on policy changes, multidisciplinary collaboration, and parental empowerment to bridge this gap and promote equitable, patient-centred care.

## Conclusion

This study conducted across two Portuguese paediatric oncology units indicates that while most parents reported receiving oral health instructions during cancer treatment, a significant gap remains in the actual implementation of oral care protocols. Oral complications were inconsistently reported, and overall parental satisfaction remained low. Furthermore, the finding that only a minimal proportion of parents implemented comprehensive oral hygiene practices suggests deficiencies in parental oral health management, potentially stemming from underlying insecurity. Future research should prioritise identifying the systemic barriers within oncology teams that hinder effective care delivery and exploring parental perspectives to enhance oral health literacy and compliance.

## Data Availability

No datasets were generated or analysed during the current study.
